# Relationship between concentration of rare earth elements in soil and their distribution in plants growing near a frequented road

**DOI:** 10.1007/s11356-018-2428-x

**Published:** 2018-06-05

**Authors:** Patrycja Mleczek, Klaudia Borowiak, Anna Budka, Przemysław Niedzielski

**Affiliations:** 1 Department of Ecology and Environmental Protection, Poznan University of Life Sciences, Piątkowska 94C, 60-649 Poznań, Poland; 2 Department of Mathematical and Statistical Methods, Poznan University of Life Sciences, Poznań, Poland; 3 Faculty of Chemistry, Adam Mickiewicz University in Poznań, Umultowska 89B, 61-614 Poznań, Poland

**Keywords:** Distribution, Frequented road, Heavy rare earth elements, Herbaceous plants, Light rare earth elements, Phytoextraction

## Abstract

Rare earth elements (REEs) are a group of elements whose concentration in numerous environmental matrices continues to increase; therefore, the use of biological methods for their removal from soil would seem to be a safe and reasonable approach. The aim of this study was to estimate the phytoextraction efficiency and distribution of light and heavy (LREEs and HREEs) rare earth elements by three herbaceous plant species: *Artemisia vulgaris* L., *Taraxacum officinale* F.H. Wigg. and *Trifolium repens* L., growing at a distance of 1, 10, and 25 m from the edge of a frequented road in Poland. The concentration of REEs in soil and plants was highly correlated (*r* > 0.9300), which indicates the high potential of the studied plant species to phytoextraction of these elements. The largest proportion of REEs was from the group of LREEs, whereas HREEs comprised only an inconsiderable portion of the REEs group. The dominant elements in the group of LREEs were Nd and Ce, while Er was dominant in the HREEs group. Differences in the amounts of these elements influenced the total concentration of LREEs, HREEs, and finally REEs and their quantities which decreased with distance from the road. According to the Friedman rank sum test, significant differences in REEs concentration, mainly between *A. vulgaris* L., and *T. repens* L. were observed for plants growing at all three distances from the road. The same relation between *A. vulgaris* L. and *T. officinale* was observed. The efficiency of LREEs and REEs phytoextraction in the whole biomass of plants growing at all distances from the road was *A. vulgaris* L. > *T. officinale* L. > *T. repens* L. For HREEs, the same relationship was recorded only for plants growing at the distance 1 m from the road. Bioconcentration factor (BCF) values for LREEs and HREEs were respectively higher and lower than 1 for all studied plant species regardless of the distance from the road. The studied herbaceous plant species were able to effectively phytoextract LREEs only (BCF > 1); therefore, these plants, which are commonly present near roads, could be a useful tool for removing this group of REEs from contaminated soil.

## Introduction

Road traffic, depending on the amount of motor vehicles, can significantly influence the contamination of particular environmental components (van Bohemen and van de Laak 2003). As a result of the ecological consequences associated with the high emission of toxic elements from traffic to the environment, phytoextraction of elements to aboveground plant organs growing near roads has begun to attract more attention. In literature, there are descriptions of the negative influence of catalytic converters responsible for the emission of platinum group elements (PGE), especially platinum (Pt), palladium (Pd), and rhodium (Rh) directly to the environment (Schäfer and Puchelt 1998; Kalavrouziotis and Koukoulakis 2009). The other pollutants emitted by vehicles are rare earth elements (REEs), present both in soil and road dust (Djingova et al. 2003; Mikołajczak et al. 2017). It is possible that the amount of vehicles may be correlated with REE concentration in soil, depending on a variety of environmental factors, especially the natural geological composition of the soils (Figueiredo et al. 2009).

A great number of studies of phytoextraction of elements in plants have been conducted (Simon et al. 1996; Swaileh et al. 2004; Jankowski et al. 2014) but they have usually focused on selected plant species and some elements only. The most commonly analyzed plants—also growing near roads—are grasses (Jankowski et al. 2015) or herbaceous plant species such as *Inula viscosa* (Swaileh et al. 2004), *Rumex acetosa* L. (Malinowska et al. 2015), or *Vicia cracca* L. (Modlingerová et al. 2012). In the above mentioned but also other papers, with the exception of PGE, the same elements (Cd, Cr, Cu, Pb, and Zn) have been analyzed in different plant species. Among numerous herbaceous species, *Taraxacum officinale* (Keane et al. 2001) and *Achillea millefolium* L. (Modlingerová et al. 2012) have been the most frequently analyzed. However, to date the phytoremediative potential of *Artemisia vulgaris* L. and *Trifolium repens* L. have been estimated only for As, Cd, Cu, Ni, Pb, and Zn (Kafoor and Kasra 2014; Çolak et al. 2016); Cu, Hg, and Pb (Pivić et al. 2013); As, Cd, Cr, Cu, Mo, Ni, Pb, and Zn (Modlingerová et al. 2012); or As, Pb and Sb (Álvarez-Ayuso et al. 2012).

In literature, there are no studies that describe the concentration of REEs in *Artemisia vulgaris* L. and *Trifolium repens* L., herbaceous plant species that commonly grow near roads. Owing to significant difficulties in the proper analysis of REEs, the majority of scientific papers have been limited to the selection of a few of them only (usually lanthanum (La) and/or neodymium (Nd)) (Diatloff et al. 2008; Lyubomirova et al. 2011; Siwulski et al. 2017). In recent years, accumulation of REEs has been mainly estimated in some plant species or in wild growing mushroom species (Mleczek et al. 2016a,b; Saatz et al. 2015; Zhang et al. 2015). Li et al. (2013) pointed out that the intake of vegetables in the vicinity of a large-scale mining area is not related with exceeding the daily intake of REEs (100–110 μg kg^1^ d^−1^) but long-term exposure to these elements in food can be a real health risk. The path of REEs accumulation together with a determination of the role of key ligands has already been established (Ding et al. 2005). The concentration of selected REEs in soil described in the studies of Ding et al. (2005), especially Ce and Nd, increased when compared to the results obtained by Ichihashi et al. (1992) or Djingova et al. (2003). It is worth underlining that increasing REEs concentration within the next few years may be associated with a major new form of environmental pollution (Li et al. 2010). Potentially, this increase may pose a threat to both plant (REEs are not nutritionally essential for plants) and human health (Thomas et al. 2014), mainly as regards the intensity of use of these elements in new technologies (rechargeable batteries, cell phones, or carbon arc lighting).

For this reason, the aim of the study was to estimate the phytoextraction efficiency of REEs in organs and whole biomass of three herbaceous plant species: *Artemisia vulgaris* L., *Taraxacum officinale* F. H. Wigg., and *Trifolium repens* L. naturally growing at three different distances (1, 10, and 25 m) from the edge of a road (traffic lane). This paper is a development of studies described in our previous studies (Mikołajczak et al. 2017) with new data about the efficiency of phytoextraction and distribution of REEs in organs of selected herbaceous plant species growing near the frequented road.

## Materials and methods

### Characteristics of experimental material and its collection

**Fig. 1:**
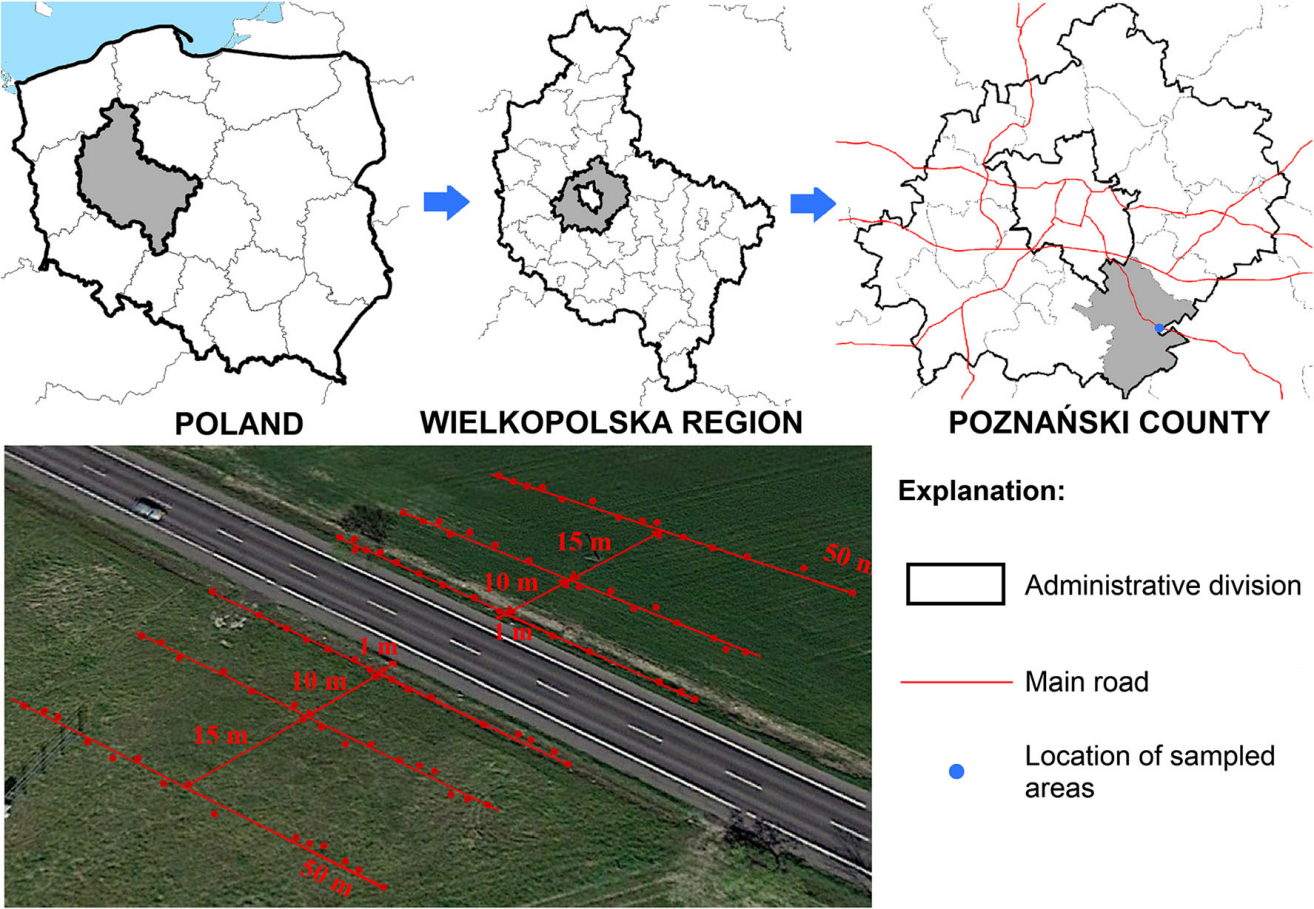
Location of experimental site and method of sample collection

Experimental materials were three herbaceous plant species: *Artemisia vulgaris* L., *Taraxacum officinale* F. H. Wigg., and *Trifolium repens* L. (Table 1), growing near the S11, a road located in the central part of the Wielkopolska Region (52° 14′ 40.07″ N 17° 07′ 28.02‶ E) (Fig. 1).

**Table 1:** Characteristics of analyzed herbaceous plant species

No.	1	2	3
Plant species	*A. vulgaris* L.	*T. officinale* F. H. Wigg.	*T. repens* L.
Common name	common wormwood, mugwort	dandelion	white clover
EPPO code	ARTVU	TAROF	TRFRE
Family	*Asteraceae*	*Asteraceae*	*Fabaceae*
Occurrence	Present at uncultivated areas, roadsides or places of wastes landfill. Asia, Europe, northern Africa, North America	The native species to Asia and Europe; present in North and South America and southern Africa and Australia	The moist temperature zones; Australasia, Europe, Japan, North America, southern Latin America,
Season of growth	Flowering between July and October	Flowering between April and July	Late spring and summer (flowering between May and November)
High [cm]	60–120	5–40	7–20
Leaves	Sessile and pinnate dark green, 5–15 cm long	Oblanceolate or obovate in shape, 3–35 cm long	Trifoliate, elliptic and smooth, 1–2 cm long

Fifteen specimens of each plant species and soil samples around the plants were collected along this road (50 m) from three distances from the edge of the road: 1, 10, and 25 m. Soil samples were collected from 0 to 15 cm depth. Based on the interpretation of the content of the soil-agricultural map, it can be concluded that the investigations were carried out on Luvisols, characterized by a loamy sand texture up to a depth of about 75 cm and sandy loam texture in the underlying horizons (IUSS Working Group WRB 2015). According to Aide and Aide (2012), REE content in this soil type is lower than other soil types; hence, the anthropogenic sources had the strongest effect on REE accumulation in soils and plants. All experimental materials were collected from two sides (north and south) of the road and twice, on 12 August, 2015, in drought conditions after a period of 13 days without rainfall, and 13 August, 2016, after some rainy days. Total rainfall within the 14 days before the plant material collection day in 2015 and 2016 was 1.2 and 27.7 mm, respectively. It is worth noting that the total rainfall between 1 June and 12 August, 2015, and 1 June and 13, August, 2016, was 176.5 and 224.7 mm, respectively, which indicates differing water conditions for plant growth. Mean temperatures within the growing season in 2015 and 2016 were 14.5 and 15.5 °C, respectively, and mean wind speed was 1.5 and 1.7 m s^−1^. The wind direction varied in the 2 years of the study. In 2015, the dominant wind directions were W, SW, E, NE, N and, NW (34.2; 12.1; 10.5; 11.4; 10.0 and 9.8%, respectively), while in 2016: W, SW, E, and NE (31.6; 15.7; 13.5; 11.4%, respectively). Meteorological data were obtained from the monitoring station of the Institute of Meteorology and Water Management in Poznań.

Whole plants were dug up using a polypropylene sample spade so as to ensure roots were not damaged (cut). All materials were transported to the laboratory immediately after plant and soil sample collection. It is the common presence of these three plant species in the vicinity of roads and the very limited data about their abilities for REE phytoextraction that makes them highly suitable for the purpose of this study.

### Preparation of samples

After transport to laboratory, each plant was carefully washed with deionized water using Milli-Q Advantage A10 Water Purification Systems, Merck Millipore (Merck, Darmstadt, Germany) to remove traffic dust (leaves and stem) and soil particles (roots). Collected plants were divided into roots, stem, and leaves, dried in an electric oven (TC 100, SalvisLAB, Switzerland) at 105 ± 3 °C for 96 h and ground for 3 min in a Cutting Mill SM 200 (Retsch GmbH, Haan, Germany). Three samples prepared for each plant organ of three herbaceous plant species were digested using the microwave mineralization system CEM Mars 5 Xpress (*CEM*, Matthews, NC, USA). Prepared samples (0.3000 ± 0.0001 g) were placed in 55 mL vessels with 8 mL of concentrated (65%) HNO_3_ Suprapur® (Merck, Darmstadt, Germany) and 1 mL of H_2_O_2_ for ultratrace analysis (Merck, Darmstadt, Germany) and digested according to a temperature program that consisted of three stages: first stage: temperature 80 °C, time 10 min, power 600 W; second stage: temperature 120 °C, time 10 min, power 1200 W; third stage: temperature 200 °C, time 12 min, power 1600 W. After digestion, the solutions were filtrated using Qualitative Filter Papers (Whatman, Grade 595 4–7 μm) and filled with deionized water Milli-Q Advantage A10 Water Purification Systems, Merck Millipore (Merck, Darmstadt, Germany) to a final volume of 50 mL. Concentrations of rare earth elements are expressed in mg kg^−1^ of dry matter (d.m.) of plant organs, both in tables and the whole text.

The preparation of soil samples followed the same procedure as the plant material. The only difference being that before separate digestion with concentrated (65%) HNO_3_ Suprapur® the samples were submitted to mercerization for 24 h and the times of particular stages of digestion were twice as long with the same temperature and power. Soil samples were also characterized by pH (PN-ISO 10390:^1997^) and redox potential (ISO 11271:^2002^) using a Microprocessor pH Meter 211 by Hanna Instruments and electrolytic conduction (PN-ISO 1265+AC1:^1997^) using an EC-meter HI 2316 by Hanna Instruments (Woonsocket, Rhode Island, USA). Chemical characteristics of soil are presented in Table 2.

**Table 2:** Characteristics of rare earth element concentration [mg kg^−1^] and selected parameters of soil collected from three distances from the edge of the road

Element	Unit	1 m	10 m	25 m
LREEs				
Ce	mg kg^−1^	18.00^a^ ± 1.23	16.31^a^ ± 1.33	8.97^b^ ± 1.01
Eu		0.15^a^ ± 0.02	0.13^a^ ± 0.01	0.08^b^ ± 0.02
Gd		1.54^a^ ± 0.15	1.31^ab^ ± 0.17	1.11^b^ ± 0.09
La		3.86^a^ ± 0.28	3.35^a^ ± 0.29	2.66^b^ ± 0.13
Nd		12.65^a^ ± 0.97	11.96^a^ ± 1.07	8.04^b^ ± 0.85
Pr		0.86^a^ ± 0.12	0.81^ab^ ± 0.09	0.67^b^ ± 0.12
Sm		0.03^a^ ± 0.01	0.03^a^ ± 0.01	0.01^b^ ± 0.00
HREEs				
Dy	mg kg^−1^	0.56^a^ ± 0.11	0.52^ab^ ± 0.09	0.41^b^ ± 0.06
Er		47.21^a^ ± 4.19	40.87^ab^ ± 4.82	36.99^b^ ± 3.61
Ho		0.04^a^ ± 0.01	0.03^a^ ± 0.01	0.01^b^ ± 0.01
Lu		0.15^a^ ± 0.03	0.13^ab^ ± 0.02	0.09^b^ ± 0.02
Sc		0.71^a^ ± 0.16	0.62^a^ ± 0.12	0.58^a^ ± 0.13
Tb		0.23^a^ ± 0.04	0.18^a^ ± 0.02	0.13^b^ ± 0.01
Tm		1.05^a^ ± 0.21	0.91^a^ ± 0.11	0.88^a^ ± 0.09
Y		2.24^a^ ± 0.25	2.03^ab^ ± 0.18	1.79^b^ ± 0.16
Yb		0.34^a^ ± 0.06	0.29^a^ ± 0.03	0.18^b^ ± 0.02
Chemical characteristics of soil				
Ca	%	0.103^a^ ± 0.009	0.099^a^ ± 0.008	0.098^a^ ± 0.011
K	%	0.116^a^ ± 0.017	0.105^a^ ± 0.011	0.078^b^ ± 0.009
Mg	%	0.105^a^ ± 0.029	0.113^a^ ± 0.015	0.102^a^ ± 0.018
Na	%	0.018^a^ ± 0.005	0.019^a^ ± 0.002	0.017^a^ ± 0.005
P	%	0.039^a^ ± 0.010	0.042^a^ ± 0.008	0.039^a^ ± 0.004
S	%	0.018^a^ ± 0.003	0.017^a^ ± 0.001	0.017^a^ ± 0.002
Fe	%	0.508^a^ ± 0.028	0.479^a^ ± 0.033	0.503^a^ ± 0.041
Mn	mg kg^−1^	131^a^ ± 19	125^a^ ± 6	128^a^ ± 9
pH	–	6.03^a^ ± 0.09	6.05^a^ ± 0.03	6.00^a^ ± 0.05
EC	μS cm^−1^	608^a^ ± 12	579^a^ ± 27	593^a^ ± 24
Eh	mV	203^ab^ ± 13	185^b^ ± 12	210^a^ ± 7

*n* = 15, mean values ± SD; identical letters (a, b, c...) followed by values denote no significant (*p* = 0.05) difference in rows (for particular element or soil parameter) according to Tukey’s HSD test (ANOVA); *bDL* below Detection Limit

The obtained results have shown a general decrease in the majority of REEs with the distance from the edge of the road (significant differences in mineral characteristics between soil samples collected from 1 and 25 m from the road). There were no significant differences between pH and EC values characterized in the studied soil samples. Significant differences were only observed in Eh values between soils collected from 10 and 25 m of the road. This was probably an effect of the shallow ground depressions found in this area, where rain water was accumulated, as well as differences in soil granulation and soil humidity, as confirmed by the different allocation of plants in this area.

### Analytical methods

The determination of REEs was carried out using inductively coupled plasma optical emission spectrometry (ICP-OES) with an Agilent 5100 (Agilent, Santa Clara, USA) spectrometer with a synchronous (dual axial and radial plasma) view. The following common instrumental parameters were used for determination of all elements: RF power 1.2 kW, plasma gas (argon) flow 12 L min^−1^, nebulizer gas (argon) flow 0.7 L min^−1^, and radial view height 8 mm. The following wavelengths were used for REE determination: Ce 446.021 nm, Dy 400.045 nm, Er 349.910 nm, Eu 420.504 nm, Gd 342.246 nm, Ho 348.484 nm, La 333.749 nm, Lu 307.760 nm, Nd 406.108 nm, Pr 417.939 nm, Sc 361.383 nm, Sm 442.434 nm, Tb 350.914 nm, Tm 336.261 nm, Y 361.104 nm, and Yb 328.937 nm. The ICP-OES instrument did not allow the determination of promethium, which is a man-made, radioactive element and is not recognized among naturally occurring lanthanides.

The detection limits were estimated at the level of 0.0X mg kg^−1^: for Ce 0.02 mg kg^−1^, 0.05 mg kg^−1^ for Dy, 0.04 mg kg^−1^ for Er, 0.07 mg kg^−1^ for Eu, 0.07 mg kg^−1^ for Gd, 0.06 mg kg^−1^ for Ho, 0.02 mg kg^−1^ for La, 0.06 mg kg^−1^ for Lu, 0.02 mg kg^−1^ for Nd, 0.06 mg kg^−1^ for Pr, 0.05 mg kg^−1^ for Sc, 0.05 mg kg^−1^ for Sm, 0.04 mg kg^−1^ for Tb, 0.06 mg kg^−1^ for Tm, 0.04 mg kg^−1^ for Y, and 0.03 mg kg^−1^ for Yb, respectively. The uncertainty was estimated on the level of 20% (*k* = 2) for the whole analytical procedure.

The certified standard material CRM NCSDC 73349 (CNACIS, Beijing, China)—bush branches and leaves was used in traceability control. The recovery values were as follows: Ce 119%, Dy 77%, Eu 77%, Gd 105%, Ho 82%, La 87%, Lu 91%, Nd 110%, Pr 83%, Sm 118%, and Yb 79%, respectively. For uncertified elements Er and Sc, an analysis of the certified standard material CRM 667 sediment (IRRM, Geel, Belgium) was additionally provided. The obtained recoveries were Er 105% and Sc 107%. Recovery values in the range of 75–125% were recognized as satisfactory.

### Statistical analysis and calculations

All statistical analyses were made using the agricole package (R). Estimation of the concentration of REEs, LREEs, or HREEs (dependent variable) in organs of herbaceous plant species (independent variable) was carried out. The mean of element concentration in particular plant species was compared. One-way analysis ANOVA with the F-Fisher test (*α* = 0.05) was used to verify the general hypothesis with respect to the equality of the mean concentration of particular LREEs or HREEs in the analyzed plant species. In the case of a null hypothesis being rejected, the Tukey test for multiple comparisons was applied to divide the studied herbaceous plant species into homogenous groups (*α* = 0.05).

For a graphical presentation of the similarities and differences between particular plant species growing at different distances from the road with respect to their phytoextraction abilities for particular LREEs or HREEs separately or all REEs, LREEs, or HREEs in the whole plant bodies, a heatmap analysis was performed. Two-dimensional variables (plant species growing at different distances and REE concentration) were represented as blue colors.

To show significant differences between tested plants as regards the concentration of all 16 REEs jointly in their whole biomass, the Friedman rank sum test was applied with pairwise comparisons using the Nemenyi multiple comparison test (posthoc.friedman.nemenyi.test) with q approximation for unreplicated blocked data. Additionally, to illustrate the potential of the analyzed plants in the phytoextraction of all 16 REE elements jointly, the rank sum was performed.

To estimate the efficiency of REE phytoextraction by the studied plant species, bioconcentration factor (BCF) values were calculated as the ratio of the concentration of HREEs, LREEs, or REEs in the harvested organs (leaves and stems) to their concentration in soil (Ali et al. 2013). Correlation coefficient (*r*) values between the concentration of LREEs, HREEs, REEs, and distances from the traffic lane were also calculated. Additionally, the concentration of REEs [mg kg^−1^] allowed the particular element contents in whole plants biomass to be calculated [mg per plant].

## Results

The results described in this paper are the mean values calculated for the parameters characterizing the materials collected in the first (2015) year of the 2-year studies (2015–2016) in the environment. The same relationships were found in the phytoextraction of LREEs, HREEs, and REEs by organs of the three studied herbaceous plant species in 2015 and 2016. With respect to the differing amount of rainfall in particular years of studies, it is likely that the differences in the level of these groups of elements were only observed.

### Data selection

**Fig. 2:**
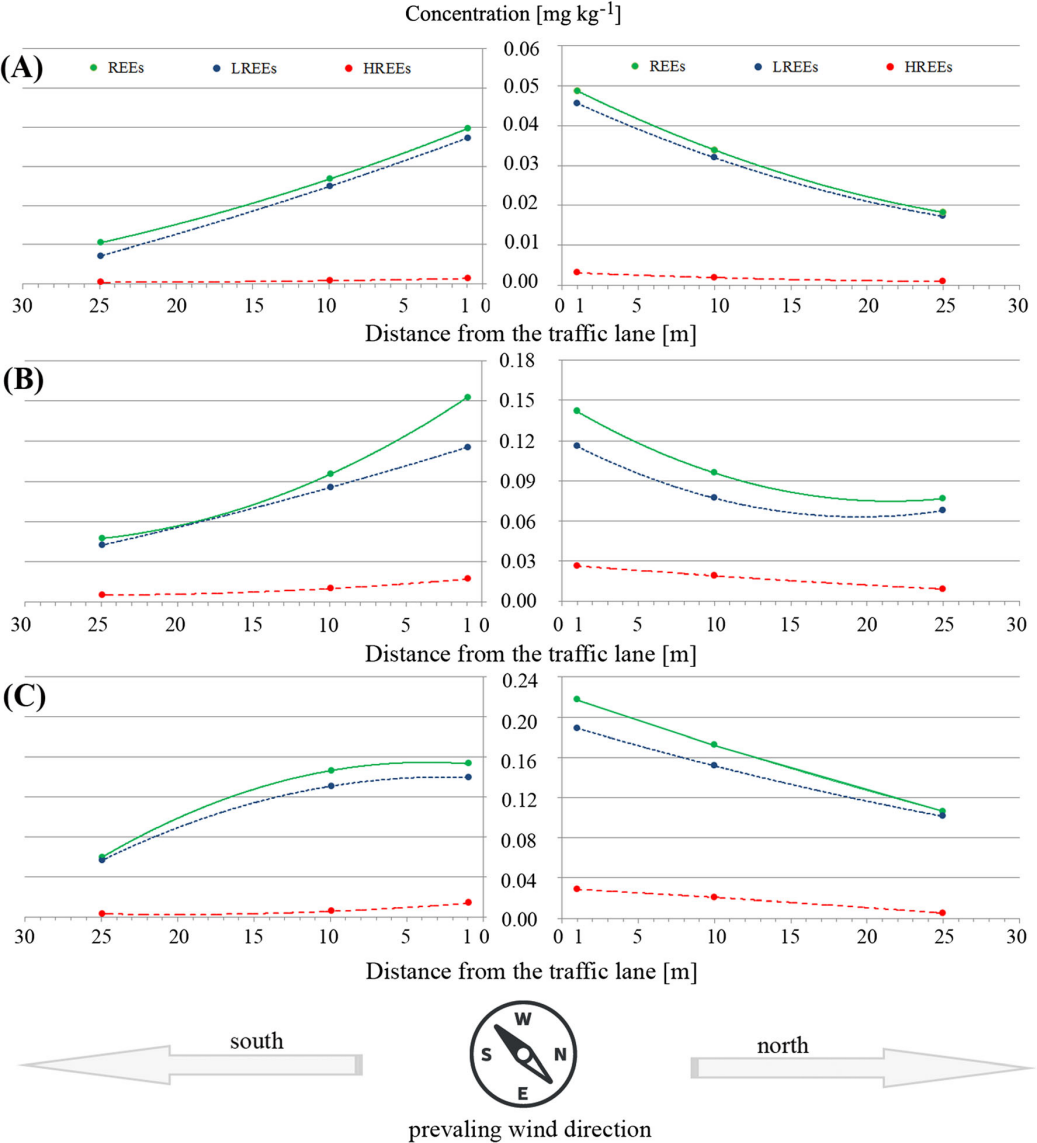
Distribution and concentration [mg kg^−1^] of REEs, LREEs, and HREEs in *Artemisia vulgaris* L. (**a**), *Taraxacum officinale* (**b**), and *Trifolium repens* L. (**c**) growing on north and south side and with respect to their distance from the traffic lane

Figure 2 presents the distribution and concentration of all three groups of elements in herbaceous plant species growing on two sides of a frequented road. The relationships between phytoextraction of LREEs and REEs were almost the same between plant species with clear differences in element concentration in plants growing on the north and south side of the road.

The dominant wind direction in the 2 years of study was west. In the case of the rest of each year, northwest and southwest winds were observed, which could suggest that plants growing on both sides of the road should be characterized by a similar concentration of REEs in their organs. We confirmed this relationship and the differences described in Fig. 2 could be an effect of variable wind directions.

In spite of the fact that in both 2015 and 2016 the same relationship of LREE, HREE, and REE phytoextraction efficiency between the studied plant species was recorded, some differences between years were clearly observed. Phytoextraction of all three groups of elements in 2015 was lower than in 2016 which was probably the effect of the better growth conditions in the last year (the higher number of rainy days). Concentration of REEs in *A. vulgaris* L. roots, stems, and leaves in 2016 was higher than that in 2015 with 13–21, 22–29, and 29–36%, respectively. In the case of the *T. officinale* and *T. repens*, these increases were 8–14 and 15–19%, respectively, in roots and 14–22 and 18–27%, respectively, in stem, while 13–18 and 20–28%, respectively, in leaves. The presentation of the 2015 results only was advisable to make the presentation of the abundant data clearer, especially as the same relationships were observed between the plants.

### Concentration of light, heavy, and total rare earth elements

The concentration of rare earth elements was diverse for organs of all three plant species growing at 1, 10, and 25 m distance from the edge of the road (Table 3). Generally, concentration of REEs, HREEs, and LREEs in plant organs decreased with the distance from the road. Significant differences (*p* = 0.05) between the concentration of these three groups of elements were recorded in selected organs of plants growing: (*i*) at distance 1 and 10 m or 25 m and also (*ii*) plants growing at a distance of 1 or 10 m and 25 m from the road. In the case of the first, the following relationship was observed for concentration of LREEs (*T. officinale* L. stem, *T. repens* L. roots and stem), HREEs (*A. vulgaris* L. roots and leaves, *T. officinale* L. roots and stem), and REEs (*A. vulgaris* L. roots; *T. officinale* roots, stems, and leaves; *T. repens* L. leaves). In the second case, a similarity of element concentration were observed for in two plants growing nearest the road with a significantly lower concentration in plants from 25 m: LREEs (*A. vulgaris* stem, *T. officinale* roots and leaves, and also *T. repens* L. roots), HREEs (*A. vulgaris* L. roots, *T. officinale* leaves and *T. repens* L. roots), and REEs (*A. vulgaris* and *T. repens* L. roots). It is worth noting that for *A. vulgaris* L. roots (LREEs), leaves (LREEs and REEs), *T. repens* L. stems (HREEs and REEs), and leaves (HREEs), significant differences between the concentration of elements in particular groups in plants growing at a distance of 1 and 25 m from the road were also observed.

**Table 3:** Concentration [mg kg^−1^ d.m.] of light, heavy and total rare earth elements in organs of herbaceous plant species in different distance from the edge of the road

Plant species	Distance from the road	Plant organ	LREEs	HREEs	REEs
*Artemisia vulgaris L.*	25 m	Root	11.26^b^ ± 1.95	0.62^b^ ± 0.10	11.88^b^ ± 2.03
	10 m		13.60^ab^ ± 1.62	1.03^b^ ± 0.10	14.63^b^ ± 1.63
	1 m		19.66^a^ ± 2.77	4.03^a^ ± 0.46	23.69^a^ ± 2.88
	F, *p* value		8.02, < 0.05	89.08, < 0.05	15.17, < 0.05
	25 m	Stem	8.82^b^ ± 0.64	0.51^b^ ± 0.16	9.33^b^ ± 0.54
	10 m		18.65^a^ ± 2.74	1.08^a^ ± 0.17	19.73^a^ ± 2.90
	1 m		26.44^a^ ± 4.09	0.98^ab^ ± 0.15	27.42^a^ ± 3.98
	F, *p* value		18.95, < 0.05	7.34, < 0.05	20.15, < 0.05
	25 m	Leaves	23.84^b^ ± 2.08	1.94^b^ ± 0.74	25.79^b^ ± 2.18
	10 m		37.38^ab^ ± 3.69	2.07^b^ ± 0.34	39.45^ab^ ± 3.36
	1 m		56.21^a^ ± 16.24	5.97^a^ ± 0.90	62.19^a^ ± 15.99
	F, *p* value		5.63, < 0.05	21.38, < 0.05	7.46, < 0.05
*Taraxacum officinale*	25 m	Root	11.46^b^ ± 1.62	1.53^b^ ± 0.28	12.99^c^ ± 1.67
	10 m		22.20^a^ ± 2.16	1.97^b^ ± 0.20	24.17^b^ ± 2.14
	1 m		27.83^a^ ± 2.88	3.08^a^ ± 0.46	30.90^a^ ± 2.59
	F, *p* value		26.59, < 0.05	11.68, < 0.05	34.87, < 0.05
	25 m	Stem	26.85^b^ ± 2.77	1.31^b^ ± 0.14	28.16^b^ ± 2.65
	10 m		31.61^b^ ± 0.89	1.99^b^ ± 0.13	33.61^b^ ± 1.00
	1 m		40.86^a^ ± 3.98	3.47^a^ ± 0.78	44.32^a^ ± 3.93
	F, *p* value		12.51, < 0.05	11.42, < 0.05	17.32, < 0.05
	25 m	Leaves	29.72^b^ ± 3.67	0.78^b^ ± 0.11	30.50^c^ ± 3.70
	10 m		45.01^a^ ± 3.75	7.98^a^ ± 1.21	52.99^b^ ± 3.91
	1 m		55.20^a^ ± 14.44	10.44^a^ ± 1.39	65.64^a^ ± 15.79
	F, *p* value		4.18, 0.07	44.34, < 0.05	6.82, < 0.05
*Trifolium repens L.*	25 m	Root	15.90^b^ ± 0.71	6.17^b^ ± 0.16	22.07^b^ ± 0.58
	10 m		38.54^a^ ± 3.83	12.45^a^ ± 1.42	50.99^a^ ± 3.13
	1 m		40.27^a^ ± 2.94	13.50^a^ ± 2.07	53.77^a^ ± 3.52
	F, *p* value		46.61, < 0.05	14.87, < 0.05	82.08, < 0.05
	25 m	Stem	30.77^b^ ± 4.73	4.78^b^ ± 1.03	35.56^b^ ± 5.67
	10 m		32.81^b^ ± 1.68	8.60^ab^ ± 2.27	41.41^ab^ ± 1.70
	1 m		39.15^a^ ± 1.88	10.29^a^ ± 1.13	49.44^a^ ± 0.75
	F, *p* value		3.99, *p* < 0.05	6.39, < 0.05	8.18, < 0.05
	25 m	Leaves	22.50^b^ ± 2.06	2.57^b^ ± 0.26	25.06^b^ ± 2.31
	10 m		25.28^b^ ± 0.99	5.88^ab^ ± 1.13	31.16^b^ ± 2.08
	1 m		42.56^a^ ± 2.93	8.88^a^ ± 2.59	51.44^a^ ± 5.23
	F, *p* value		51.44, < 0.05	7.44, < 0.05	30.92, < 0.05

*n* = 15, mean values ± SD; identical letters (a, b, c...) followed by values denote no significant (*p* = 0.05) difference in columns (for particular organs and plant species) according to Tukey’s HSD test (ANOVA)

LREEs formed the main component of REEs concentration (significantly lower concentration of HREEs than that of LREEs in the sum of REEs). For *A. vulgarsis* L. and *T. officinale*, the highest concentration of LREEs, HREEs, and REEs was observed in leaves while for *T. repens* L. the concentration of all element groups was more uniform in the whole plant biomass.

To show the efficiency of REE phytoextraction, bioconcentration factor (BCF) values were calculated (Table 4). Only for LREEs, was the BCF > 1 observed for all plant species, regardless of distance from the edge of the road. The opposite situation was recorded for HREEs, while in the case of the REEs, BCF > 1 was found for all plant species growing at the 1-m distance from the road (from BCF = 1.23 for *T. officinale* to BCF = 1.01 for *A. vulgaris* L.) and also *T. officinale* growing 10 m from the road only (BCF = 1.09). Moreover, a decrease of the BCF with an increase of the distance from the road was observed for phytoextraction of LREEs, HREEs and REEs by *A. vulgaris* L., HREEs and REEs by *T. officinale*, and also HREEs by *T. repens* L.

**Table 4:** Bioconcentration factor (BCF) values of LREEs, HREEs, and REEs with correlation coefficient (*r*) values

Plant species	Distance from the road	LREEs	HREEs	REEs
*A. vulgaris L*	25 m	1.52	0.06	0.56
	10 m	1.65	0.07	0.74
	1 m	2.23	0.13	1.01
r		0.9338	0.9672	0.9780
*T. officinale*	25 m	2.63	0.05	0.94
	10 m	2.26	0.22	1.09
	1 m	2.59	0.26	1.23
r		0.9495	0.9515	0.9958
*T. repens L.*	25 m	2.47	0.18	0.97
	10 m	1.71	0.32	0.91
	1 m	2.20	0.36	1.13
*r*		0.7695	0.9711	0.9311

### Concentration of particular light and heavy rare earth elements

Characteristics of LREEs in the organs of the three herbaceous plant species are presented in Table 5. There were no significant differences between the concentration of these elements in plant organs and the distance from the road, especially for *A. vulgaris* L. (Pr in roots; Eu and Sm in stems; and also Ce, Eu, Pr, and Sm in leaves); *T. officinale* (Ce, Eu, Gd, and La in roots; Ce, Eu, Gd, La, and Sm in stems; and also Ce, Eu, Pr, and Sm in leaves); and *T. repens* L. (Pr in roots; Ce, Eu, Nd, Pr, and Sm in stems; and Ce, Eu, Gd, and La in leaves). A significant difference between the concentration of selected LREEs in plant organs from 1 m and 10 or 25 m were observed for Ce, Eu, and Sm in *A. vulgaris* roots; Nd and La in stems and leaves, respectively. The same relationships were recorded for Gd in stems and Nd in leaves of *T. repens* L. Additionally, a similar concentration of particular LREEs in organs of plants from a distance of 1 and 10 m from the road with simultaneous significantly lower concentration in plants from 25 m were observed for Gd in *A. vulgaris* L. leaves, Nd and Pr in roots and Nd in *T. officinale* leaves, and also Ce, Gd, La, and Nd in *T. repens* L. roots.

**Table 5:** Concentration [mg kg^−1^] of particular light rare earth elements in organs of herbaceous plant species in different distance from the edge of the road

Plant species	Distance from the road	Plant organ	Ce	Eu	Gd	La	Nd	Pr	Sm
*Artemisia vulgaris L.*	25 m	Root	2.18^b^	0.04^b^	0.11^ab^	0.34^b^	7.54^b^	1.05^a^	0.01^b^
	10 m		1.90^b^	0.04^b^	0.15^a^	0.86^a^	9.85^ab^	0.79^a^	0.01^b^
	1 m		5.51^a^	0.08^a^	0.04^b^	0.26^b^	13.03^a^	0.71^a^	0.04^a^
	F*, p* value		13.67 < 0.05	8.00 < 0.05	6.59 < 0.05	12.16 < 0.05	5.04 < 0.05	1.78 0.25	9.14 < 0.05
	25 m	Stem	1.61^b^	0.04^a^	0.15^a^	0.08^b^	6.71^b^	0.23^b^	0.01^a^
	10 m		4.65^a^	0.04^a^	0.08^b^	0.15^a^	12.90^b^	0.83^a^	0.01^a^
	1 m		3.15^ab^	0.08^a^	0.04^b^	0.08^b^	23.03^a^	0.04^b^	0.04^a^
	F, *p* value		18.35 < 0.05	4.80 0.06	18.98 < 0.05	8.20 < 0.05	16.07 < 0.05	43.47 < 0.05	4.92 0.05
	25 m	Leaves	5.06^a^	0.08^a^	0.04^b^	0.19^b^	17.61^b^	0.86^a^	0.01^a^
	10 m		5.08^a^	0.04^a^	0.15^a^	0.51^b^	30.79^ab^	0.81^a^	0.01^a^
	1 m		6.53^a^	0.08^a^	0.15^a^	0.68^a^	47.55^a^	1.20^a^	0.04^a^
	F, *p* value		0.69 < 0.05	4.36 0.07	17.25 < 0.05	5.29 < 0.05	6.09 < 0.05	1.75 0.25	4.92 0.05
*Taraxacum officinale*	25 m	Root	3.19^a^	0.04^a^	0.08^a^	0.49^a^	6.95^b^	0.71^b^	0.01^b^
	10 m		4.54^a^	0.04^a^	0.08^a^	0.49^a^	15.94^a^	1.09^a^	0.04^ab^
	1 m		5.18^a^	0.08^a^	0.08^a^	0.56^a^	20.55^a^	1.31^a^	0.08^a^
	F, *p* value		2.83 0.14	3.43 0.10	0.00 1.00	0.71 0.53	23.41 < 0.05	2.83 0.14	10.48 < 0.05
	25 m	Stem	4.99^a^	0.04^a^	0.04^a^	0.26^a^	20.20^b^	1.31^a^	0.01^a^
	10 m		4.80^a^	0.04^a^	0.04^a^	0.30^a^	25.91^ab^	0.49^b^	0.04^a^
	1 m		6.11^a^	0.08^a^	0.08^a^	0.34^a^	32.72^a^	1.50^a^	0.04^a^
	F, *p* value		1.32 0.34	6.00 < 0.05	3.69 0.09	0.33 0.73	13.44 < 0.05	12.10 < 0.05	4.57 0.06
	25 m	Leaves	4.73^a^	0.02^a^	0.04^b^	0.23^b^	23.24^b^	1.46^a^	0.01^a^
	10 m		5.74^a^	0.04^a^	0.08^ab^	0.41^ab^	37.58^a^	1.13^a^	0.04^a^
	1 m		5.85^a^	0.04^a^	0.11^a^	0.45^a^	46.80^a^	1.88^a^	0.08^a^
	F, *p* value		1.10 0.39	0.59 0.58	5.75 < 0.05	6.92 < 0.05	3.71 0.09	2.43 0.17	1.73 0.26
*Trifolium repens L.*	25 m	Root	4.43^b^	0.04^b^	0.08^b^	0.34^b^	10.31^b^	0.64^a^	0.08^a^
	10 m		9.64^a^	0.11^a^	0.64^a^	1.99^a^	25.21^a^	0.94^a^	0.02^b^
	1 m		10.84^a^	0.08^ab^	0.60^a^	2.21^a^	25.31^a^	1.20^a^	0.04^ab^
	F*, p* value		9.77 < 0.05	10.18 < 0.05	8.93 < 0.05	14.27 < 0.05	24.13 < 0.05	3.98 0.08	7.16 < 0.05
	25 m	Stem	5.25^a^	0.04^a^	0.04^c^	0.26^b^	24.25^a^	0.86^a^	0.08^a^
	10 m		6.15^a^	0.08^a^	0.23^b^	0.71^ab^	23.85^a^	1.76^a^	0.04^a^
	1 m		7.84^a^	0.04^a^	0.38^a^	1.24^a^	27.60^a^	2.03^a^	0.04^a^
	F, *p* value		4.09 0.08	6.00 < 0.05	25.87 < 0.05	9.92 < 0.05	0.92 < 0.45	2.90 0.13	2.67 0.15
	25 m	Leaves	4.65^a^	0.04^a^	0.04^a^	0.15^a^	17.02^b^	0.49^b^	0.11^a^
	10 m		6.04^a^	0.04^a^	0.15^a^	0.64^a^	17.51^b^	0.86^ab^	0.05^b^
	1 m		7.05^a^	0.08^a^	0.23^a^	1.13^a^	32.93^a^	1.13^a^	0.04^b^
	F, *p* value		2.32 0.18	1.71 0.26	3.17 0.12	4.16 0.07	19.58 < 0.05	10.06 < 0.05	9.77 < 0.05

*n* = 15, mean values ± SD; identical letters (a, b, c...) followed by values denote no significant (*p* = 0.05) difference in columns (for particular organs and plant species) according to Tukey’s HSD test (ANOVA)

In the rest of cases, concentrations of LREEs in plant organs were usually significantly different between plants growing at 1 and 15 m, while there were no significantly different concentrations between plants from 1 and 10 m or 10 and 25 m. It is worth emphasizing that the concentration of Gd in *A. vulgaris* L. stem, Pr in *T. officinale* stem, and Sm in *T. repens* L. roots and leaves was significantly higher in plants growing at 25 m than 10 m with similar values to plants growing 1 m from the edge of the road. This observation suggests that particular LREEs are accumulated and transported to aerial parts in a way that is characteristic for these plants, which is generally not related to the same trend observed for the sum of LREEs (decrease of their phytoextraction with distance from the road).

Differences in LREEs concentration was especially visible in the roots of *A. vulgaris* L. and *T. repens* L., although no significant differences were observed between the concentrations of Pr in plants growing at different distances from the road. The highest concentration of LREEs was stated for Nd. Only for this element was significant differences observed for all organs of the three plant species in relation to distance from the road, with the exception of *T. repens* L. stems. Nd and Ce were two dominant LREEs present in the tested plant species.

No significant differences in concentration of Ho, Lu, Tb, and Yb in organs of all plants growing at 1, 10, and 25 m from the road were observed with the exception of Tb in *A. vulgaris* L. leaves and *T. officinale* roots and also Yb in *A. vulgaris* L. stems and *T. repens* L. leaves. For Dy, Sc, and Tm, there were no significant differences for *A. vulgaris* L. and *T. officinale* organs collected from particular distances from the road. The same relationships for Y concentration in *A. vulgaris* L. stems and *T. officinale* roots and stems were recorded. A dominant HREE characterized by significantly higher concentration in the studied plant species organs growing at a distance of 1 m from the road was Er. Concentration of Er was generally uniform in *T. repens* L. organs, whereas in the other plant species, this element was effectively transported to the leaves. In the group of HREEs, the lowest diversity was observed for Ho, Lu, Tm, Yb, Sc, and Tb, mainly in *T. officinale* and *T. repens* L. organs. For this reason, it is interesting to note that the tendency described for HREEs related with a decrease in their concentration in plant organs with the distance from the road was an effect of Er concentration.

### Comparison of REEs phytoextraction in whole plant biomass

Estimation of phytoextraction efficiency can be confirmed by taking plant biomass into consideration to show how great an amount of element was accumulated in the whole plant biomass (Table 6).

**Table 6:** Concentration [mg kg^−1^] of particular heavy rare earth elements in organs of herbaceous plant species in different distance from the edge of the road

Plant species	Distance from the road	Plant organ	Dy	Er	Ho	Lu	Sc	Tb	Tm	Y	Yb
*Artemisia vulgaris L*	25 m	Root	0.01^a^	0.30^b^	0.01^a^	0.01^a^	0.04^a^	0.01^a^	0.04^a^	0.19^b^	0.01^a^
	10 m		0.01^a^	0.64^b^	0.04^a^	0.04^a^	0.04^a^	0.01^a^	0.04^a^	0.19^b^	0.04^a^
	1 m		0.01^a^	3.12^a^	0.04^a^	0.04^a^	0.08^a^	0.04^a^	0.11^a^	0.56^a^	0.04^a^
	F, *p* value		0.00 1.00	35.05 < 0.05	1.56 0.28	0.96 0.43	3.69 0.09	2.56 0.16	4.90 0.05	3.55 0.10	1.68 0.26
	25 m	Stem	0.01^a^	0.34^b^	0.02^a^	0.03^a^	0.04^a^	0.01^a^	0.04^a^	0.02^a^	0.01^b^
	10 m		0.01^a^	0.75^a^	0.04^a^	0.08^a^	0.04^a^	0.02^a^	0.04^a^	0.04^a^	0.08^a^
	1 m		0.01^a^	0.60^ab^	0.04^a^	0.08^a^	0.04^a^	0.04^a^	0.04^a^	0.05^a^	0.09^a^
	F, *p* value		0.00 1.00	6.71 < 0.05	0.78 0.50	3.41 0.10	0.00 1.00	3.06 0.12	0.00 1.00	1.34 0.33	17.71 < 0.05
	25 m	Leaves	0.01^a^	1.73^b^	0.01^a^	0.02^a^	0.04^a^	0.02^b^	0.04^a^	0.08^b^	0.01^a^
	10 m		0.01^a^	1.50^b^	0.04^a^	0.04^a^	0.06^a^	0.02^b^	0.08^a^	0.30^a^	0.04^a^
	1 m		0.01^a^	5.18^a^	0.04^a^	0.04^a^	0.08^a^	0.08^a^	0.08^a^	0.45^a^	0.04^a^
	F *p* value		0.00 1.00	19.60 < 0.05	4.57 0.06	0.78 0.50	2.77 0.14	18.06 < 0.05	1.92 0.23	29.32 < 0.05	3.20 0.11
*Taraxacum officinale.*	25 m	Root	0.01^a^	1.01^b^	0.01^a^	0.04^a^	0.04^a^	0.01^b^	0.08^a^	0.30^a^	0.04^a^
	10 m		0.01^a^	1.50^ab^	0.04^a^	0.04^a^	0.04^a^	0.01^b^	0.04^a^	0.26^a^	0.04^a^
	1 m		0.01^a^	2.37^a^	0.04^a^	0.06^a^	0.08^a^	0.04^a^	0.08^a^	0.38^a^	0.04^a^
	F, *p* value		0.00 1.00	8.25 < 0.05	2.46 0.17	1.40 0.32	3.43 0.10	64.00 < 0.05	1.37 0.32	1.74 0.25	0.01 1.00
	25 m	Stem	0.01^a^	1.01^b^	0.01^a^	0.04^a^	0.04^a^	0.01^b^	0.04^a^	0.11^a^	0.04^a^
	10 m		0.01^a^	1.58^b^	0.04^a^	0.06^a^	0.04^a^	0.01^b^	0.04^a^	0.19^a^	0.04^a^
	1 m		0.01^a^	2.98^a^	0.04^a^	0.06^a^	0.04^a^	0.04^a^	0.08^a^	0.19^a^	0.04^a^
	F, *p* value		0.00 1.00	11.64 < 0.05	4.57 0.06	1.00 0.42	0.00 1.00	1.23 0.36	1.23 0.36	1.25 0.35	0.02 0.98
	25 m	Leaves	0.01^a^	0.45^b^	0.01^a^	0.04^a^	0.08^a^	0.01^a^	0.04^a^	0.11^b^	0.04^a^
	10 m		0.01^a^	7.50^a^	0.04^a^	0.05^a^	0.08^a^	0.01^a^	0.04^a^	0.23^ab^	0.04^a^
	1 m		0.01^a^	9.79^a^	0.04^a^	0.08^a^	0.08^a^	0.04^a^	0.08^a^	0.26^a^	0.08^a^
	F, *p* value		0.00 1.00	44.10 < 0.05	2.46 0.17	2.18 0.19	0.00 1.00	4.92 0.05	1.50 0.30	7.44 < 0.05	2.67 0.15
*Trifolium repens L.*	25 m	Root	0.05^b^	5.63^b^	0.04^a^	0.04^a^	0.04^b^	0.04^a^	0.11^b^	0.19^b^	0.04^a^
	10 m		0.11^ab^	10.02^a^	0.03^a^	0.08^a^	0.34^a^	0.04^a^	0.38^a^	1.43^a^	0.04^a^
	1 m		0.15^a^	11.06^a^	0.04^a^	0.08^a^	0.30^a^	0.08^a^	0.38^a^	1.35^a^	0.08^a^
	F, *p* value		10.37 < 0.05	8.81 < 0.05	0.02 0.98	4.00 0.08	36.71 < 0.05	1.60 0.28	7.12 < 0.05	60.63 < 0.05	1.55 0.29
	25 m	Stem	0.02^b^	4.35^b^	0.04^a^	0.04^a^	0.04^b^	0.04^a^	0.08^b^	0.15^b^	0.04^a^
	10 m		0.04^b^	7.05^ab^	0.05^a^	0.04^a^	0.19^a^	0.04^a^	0.26^a^	0.90^a^	0.04^a^
	1 m		0.14^a^	9.25^a^	0.04^a^	0.04^a^	0.11^ab^	0.04^a^	0.11^b^	0.49^ab^	0.08^a^
	F, *p* value		8.60 < 0.05	5.85 < 0.05	0.22 0.81	0.00 1.00	10.13 < 0.05	0.00 1.00	13.54 < 0.05	11.15 < 0.05	1.92 0.23
	25 m	Leaves	0.02^b^	2.29^b^	0.04^a^	0.04^a^	0.04^b^	0.04^a^	0.04^a^	0.04^c^	0.04^b^
	10 m		0.04^b^	4.88^ab^	0.03^a^	0.04^a^	0.15^a^	0.04^a^	0.15^a^	0.41^b^	0.15^a^
	1 m		0.14^a^	7.65^a^	0.04^a^	0.04^a^	0.08^ab^	0.04^a^	0.11^a^	0.64^a^	0.15^a^
	F, *p* value		13.40 < 0.05	7.10 < 0.05	0.11 0.90	0.11 0.99	5.65 < 0.05	0.00 1.00	4.20 0.07	6.37 < 0.05	7.66 < 0.05

*n* = 15, mean values ± SD; identical letters (a, b, c...) followed by values denote no significant (*p* = 0.05) difference in columns (for particular organs and plant species) according to Tukey’s HSD test (ANOVA)

**Fig. 3:**
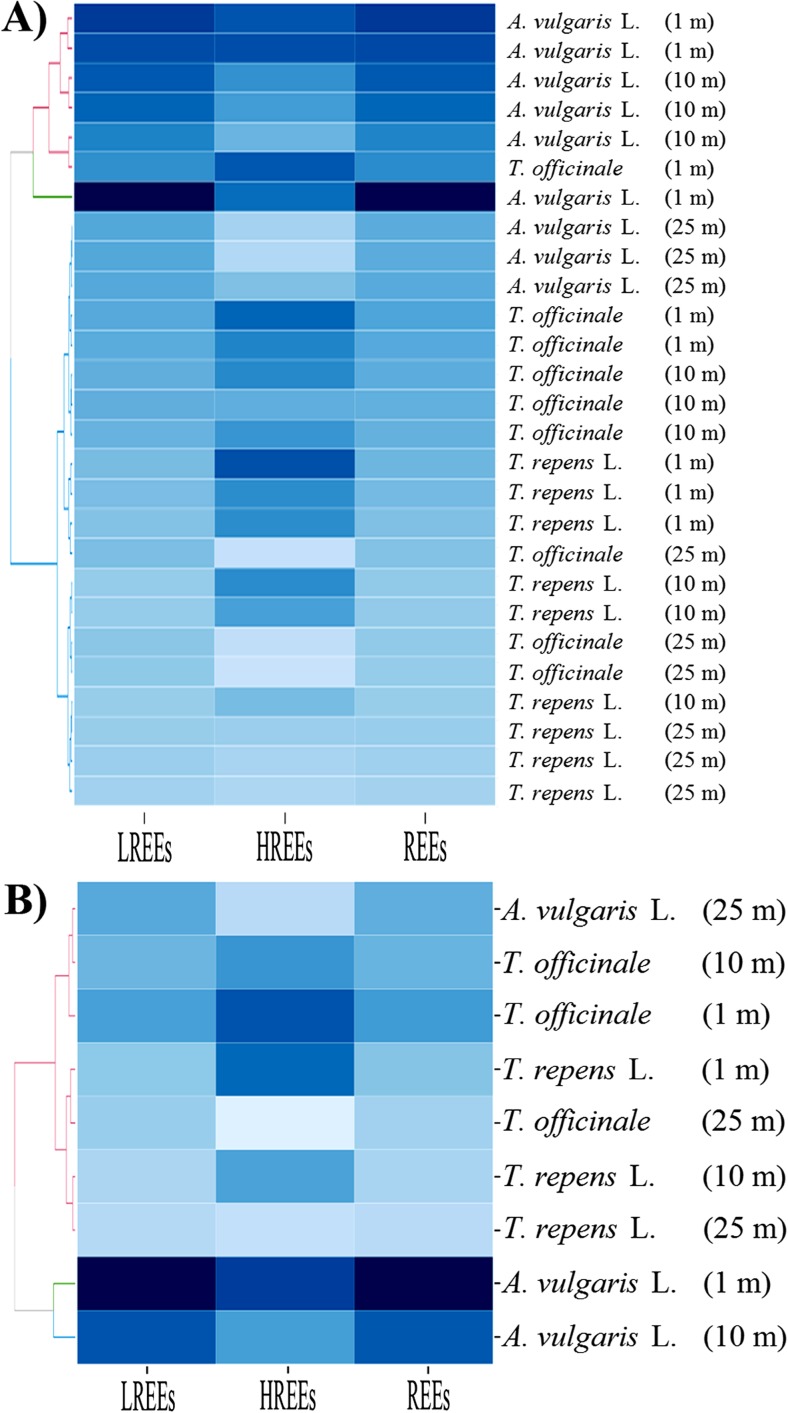
Correlation between herbaceous plant species collected from three distances from the road with respect to the concentration of REEs, HREEs and LREEs (Heatmap) in all collected specimens (**a**) and the mean values (**b**) with presentation of a hierarchical tree plot

The data presented in Table 6 confirm that the content of elements belonging to LREEs, HREEs and REEs decreased with the distance from the road. It is worth emphasizing that the results which show the content of REEs and LREEs in plants growing at 1, 10, and 25 m from the road suggest that phytoextraction efficiency for these element groups was as follows: *A. vulgaris* L. > *T. officinale* ≥ *T. repens* L. In the case of HREEs, the same relationships were not observed, thus confirming the data presented in the heatmap (Fig. 3).

**Fig. 4:**
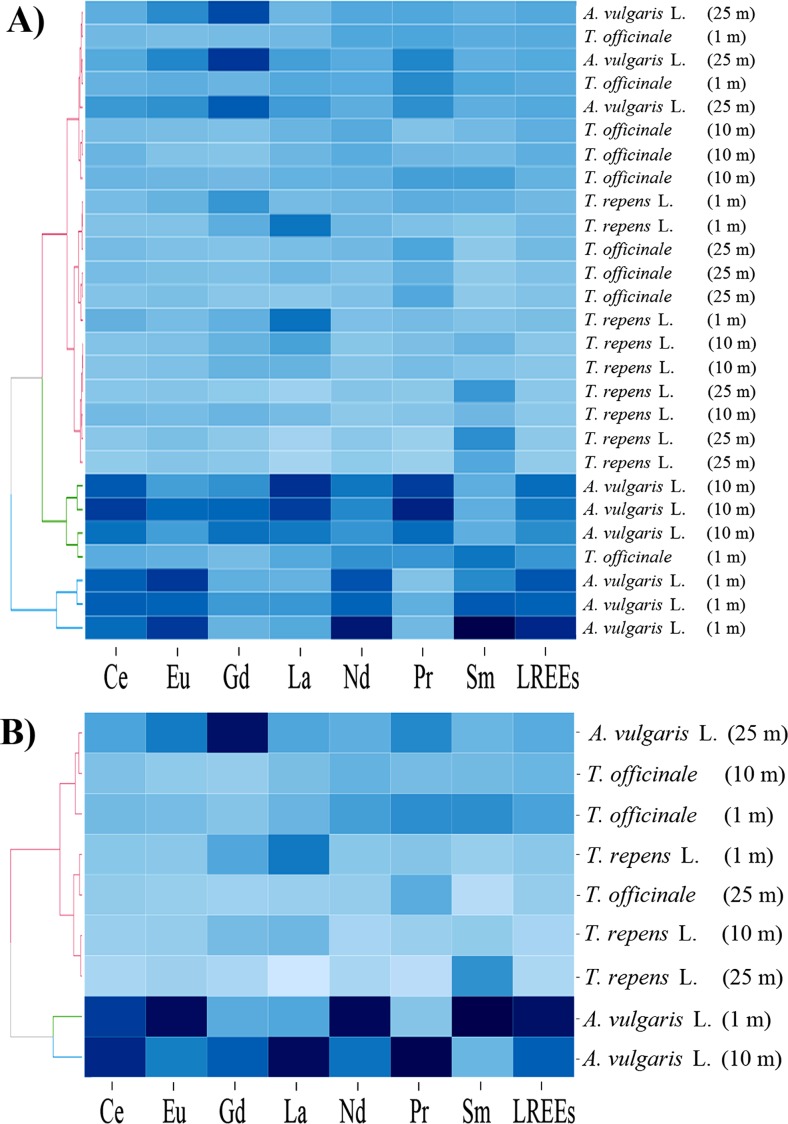
Correlation between herbaceous plant species collected from three distances from the road with respect to the concentration of particular LREEs (Heatmap) in all collected specimens (**a**) and the mean values (**b**) with presentation of a hierarchical tree plot

**Fig. 5:**
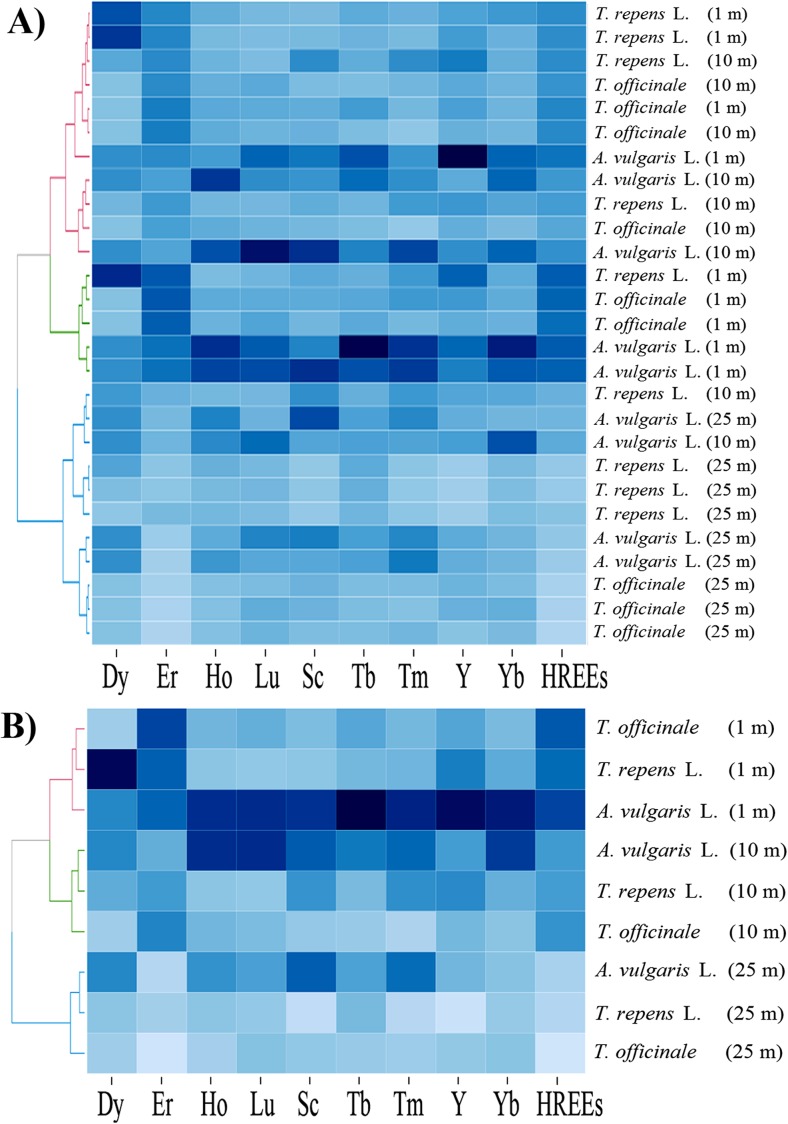
Correlation between herbaceous plant species collected from three distances from the road with respect to the concentration of particular HREEs (Heatmap) in all collected specimens (**a**) and the mean values (**b**) with presentation of a hierarchical tree plot

It is interesting to note that the color intensity (the darker the higher the concentration of elements) of particular rectangles of both specific plants (Fig. 3a) and mean values (Fig. 3b) for LREEs and REEs was the same, which would indicate that for REEs the largest part of these elements is LREEs, whereas HREEs comprise only an inconsiderable portion of the REEs. Characteristics of the heatmaps prepared separately for LREEs and HREEs are shown in Figs. 4 and 5.

The Friedman rank sum test determined some significant differences with respect to the content of LREEs, HREEs, and REEs between the compared plant species growing at varying distances from the road. In the case of LREEs, significant differences were only observed between *A. vulgaris* L. and *T. repens* L. growing 1 m from the road (Friedman chi-squared (χ_F_^2^) = 8.8571; *p* value = 0.0119). For HREEs, significant differences between plant species growing at all three distances were observed. In the case of 25 m, significant differences between *A. vulgaris* L. and *T. repens* L. were confirmed (χ_F_^2^ = 7.5152, *p* value = 0.0233). At 1 and 10 m, significant differences between *T. officinale* and *A. vulgaris* L. and also between *A. vulgaris* L. and *T. repens* L. were observed (χ_F_^2^ = 8.0741, *p* value = 0.0177 and χ_F_^2^ = 13.273, *p* value = 0.00131, respectively, for plants collected from 1 and 10 m from the edge of the road).

Significant differences between REEs content in *A. vulgaris* L. and *T. repens* (χ_F_^2^ = 6.7458, *p* value = 0.0343) growing at a distance of 25 m from the road were noted. For plants growing 10 m from the road, there were significant differences between *A. vulgaris* L. and *T. repens* L. and also *A. vulgaris* L. and *T. officinale* (χ_F_^2^ = 18.931, *p* value = 7.748e^−5^). Differences between the same herbaceous plant species growing in direct proximity to the road (1 m) were also observed (χ_F_^2^ = 16.036, *p* value = 0.000329).

In order to define the efficiency of particular analyzed herbaceous plant species in the phytoextraction of LREEs, HREEs, and REEs, the rank sum was performed. According to this analysis, the efficiency of LREE and REE phytoextraction in all tested plant bodies was as follows: *A. vulgaris* L. > *T. officinale* > *T. repens* L. In the case of HREEs, the same relationship was only observed for plants growing at a distance of 1 m.

To show the relationship between the concentration of LREEs, HREEs, and REEs in soil and their total content in the studied plant species, the correlation coefficient factor values were calculated (Table 7). With the exception of the LREEs in the whole biomass of *T. repens* (*r* = 0.7695), all *r* values were higher than 0.93, which interchangeably has shown that the concentration of these elements in soil plays a significant role in phytoextraction of all three groups of elements by the analyzed plant species.

**Table 7:** Content [mg per plant] of rare earth elements in whole herbaceous plant species growing in three distances from the edge of the road

Plant species	Distance from the road	Ce	Eu	Gd	La	Nd	Pr	Sm	LREEs	REEs	
*A. vulgaris L.*	25 m	0.03229^b^	0.00068^b^	0.00245^a^	0.00235^b^	0.12702^b^	0.00734^b^	0.00018^b^	0.17231^b^	0.18242^c^	
	10 m	0.07203^a^	0.00066^b^	0.00164^b^	0.00546^a^	0.22543^b^	0.01440^a^	0.00018^b^	0.31980^a^	0.33918^b^	
	1 m	0.06601^a^	0.00132^a^	0.00072^b^	0.00233^b^	0.38167^a^	0.00376^b^	0.00066^a^	0.45647^a^	0.48759^a^	
*T. officinale*	25 m	0.01877^a^	0.00013^a^	0.00022^a^	0.00141^a^	0.07547^c^	0.00519^b^	0.00004^c^	0.10123^b^	0.10625^b^	
	10 m	0.02256^a^	0.00016^a^	0.00029^a^	0.00181^a^	0.12239^b^	0.00428^b^	0.00016^b^	0.15166^a^	0.17223^b^	
	1 m	0.02496^a^	0.00026^a^	0.00040^a^	0.00203^a^	0.15387^a^	0.00706^a^	0.00029^a^	0.18887^a^	0.21733^a^	
*T. repens L.*	25 m	0.01331^a^	0.00010^a^	0.00011^b^	0.00051^c^	0.05155^b^	0.00163^b^	0.00028^a^	0.06750^b^	0.07656^b^	
	10 m	0.01724^a^	0.00014^a^	0.00052^ab^	0.00197^b^	0.05385^b^	0.00303^ab^	0.00012^b^	0.07686^b^	0.09579^b^	
	1 m	0.02054^a^	0.00018^a^	0.00077^a^	0.00332^a^	0.08696^a^	0.00376^a^	0.00010^b^	0.11563^a^	0.14176^a^	
Plant species	Distance from the road	Dy	Er	Ho	Lu	Sc	Tb	Tm	Y	Yb	HREEs
*A. vulgaris L.*	25 m	0.00018^a^	0.00653^c^	0.00031^a^	0.00040^a^	0.00066^a^	0.00018^b^	0.00066^a^	0.00102^b^	0.00018^b^	0.01011^c^
	10 m	0.00018^a^	0.01320^b^	0.00066^a^	0.00116^a^	0.00067^a^	0.00032^b^	0.00068^a^	0.00135^b^	0.00116^a^	0.01938^b^
	1 m	0.00018^a^	0.02224^a^	0.00066^a^	0.00116^a^	0.00082^a^	0.00068^a^	0.00096^a^	0.00304^a^	0.00138^a^	0.03113^a^
*T. officinale*	25 m	0.00004^a^	0.00333^b^	0.00004^b^	0.00016^a^	0.00024^a^	0.00004^b^	0.00022^a^	0.00077^b^	0.00017^a^	0.00502^c^
	10 m	0.00004^a^	0.01853^a^	0.00016^a^	0.00021^a^	0.00024^a^	0.00004^b^	0.00016^a^	0.00101^ab^	0.00016^a^	0.02057^b^
	1 m	0.00004^a^	0.02567^a^	0.00016^a^	0.00030^a^	0.00029^a^	0.00016^a^	0.00033^a^	0.00124^a^	0.00024^a^	0.02845^a^
*T. repens L.*	25 m	0.00006^b^	0.00814^b^	0.00010^a^	0.00010^a^	0.00010^b^	0.00010^a^	0.00014^b^	0.00020^b^	0.00010^b^	0.00906^b^
	10 m	0.00011^b^	0.01560^ab^	0.00010^a^	0.00011^a^	0.00046^a^	0.00010^a^	0.00052^a^	0.00159^b^	0.00033^a^	0.01893^ab^
	1 m	0.00039^a^	0.02272^a^	0.00010^a^	0.00011^a^	0.00026^ab^	0.00011^a^	0.00034^ab^	0.00174^a^	0.00036^a^	0.02613^a^

*n* = 15, mean values ± SD; identical letters (a, b, c...) followed by values denote no significant (*p* = 0.05) difference in columns (for particular plant species from different distance from the road) according to Tukey’s HSD test (ANOVA)

## Discussion

The presence of trace toxic elements in the environment is a real ecological problem that can have a harmful influence on living organisms (Li et al. 2010; Pagano et al. 2015b). REEs are not described as potentially toxic for humans but their increasing use in new technologies and consequent transport to the environment may eventually lead to dangerous levels of concentration (Mleczek et al. 2017). Roads are only one of the sources of REEs (Kennedy and Mitchell Limited 2003), but owing to the high charge of pollutants that may be transferred to soil adjacent to roads (Djingova et al. 2003), it is necessary to find a definite solution to reduce the amount of REEs that accumulate nearby. One of the most promising ways is the phytoextraction of pollutants by plants growing near the road. Herbaceous plant species seem to be highly suitable for such purpose not only because of their common presence near roads but also because of their dense growth in population per area unit. Phytoextraction efficiency depends on many environmental factors, but we have shown that thanks to the high correlation coefficient values (*r* > 0.9300) between REE concentration in soil and plant organs, the concentration of these elements has—together with their concentration in road dust—a decisive influence on their accumulation in the studied plants. The same observation was described by Carpenter et al. (2015), who have shown that phytoextraction of REEs increases in plant organs (especially in roots) with an increase of their concentration in soil. This relationship was shown by a hydroponic experiment of Saatz et al. (2015), where low concentrations of Gd and Y (0.1 and 1 mg L^−1^) or a higher concentration of these metals (10 mg L^−1^) used in nutritional solution were respectively unrelated with a negative, or were the cause of insignificant influence to plant biomass. Moreover, the response of plants growing under REEs depends on their concentration in soil and the kind of substrate (hydroponic, soil, wastes). We have also shown that REEs were accumulated mainly in roots but also in leaves of the studied herbaceous plant species which indicates the high potential of these elements for phytoextraction and translocation to aerial plant parts (Saatz et al. 2015). A plant characterized by high efficiency of REEs phytoextraction when growing in a hydroponic experiment, *Zea mays* studied by Saatz et al. (2016) was found to activate specific defense mechanisms. The authors reported an extremely high concentration of Gd and Y (3.17 and 8.43 g kg^−1^, respectively) in the roots of the maize, which was gained thanks to the accumulation of these elements at the epidermis thereby limiting the availability of REEs and increasing the plants’ survivability.

One problem of phytoextraction of REEs is usually related with plant species and the amount of element concentrations in substrates (Zhuang et al. 2017), because only selected plants are suitable for this purpose, as confirmed by Zhang and Shan (2001) after fertilizer application. Chemical characteristics of substrate and plant species can modulate its response and phytoextraction of REEs (Saatz et al. 2015). An example can be differences in our observation in relation to the studies of Agnan et al. (2014), who have analyzed numerous lichen and moss species. The ability of these plants to phytoextract particular elements was as follows: Ce > La > Nd > Pr > Sm > Gd > Dy > Er > Yb > Eu > Tb > Ho > Tm > Lu. The same relationship between element concentration (La > Nd > Gd > Er) was described by Wiche et al. (2017), who have analyzed REEs in *Brassica napus*, *Hordeum vulgare*, and *Zea mays*. They have shown that the amount of bioavailable REEs is about 30% of their total concentration in the soil, and the phytoextraction efficiency of these elements by plants can differ in moist and mesic grassland. This could explain the difference of these observations in relation to our studies, where for the weed species, the efficiency of REE phytoextraction was Nd > Ce > Er > La> other elements, with some exceptions, dependent on the distance from the road and the plant species. The stated differences could be the result of the varying bioavailability of elements in soil near the frequented road or the total element concentration in soils (Abechi et al. 2010; Adedeji et al. 2013). Carpenter et al. (2015) described different phytoextraction of particular REEs as regards their concentration in soil, e.g., Nd > Pr > r or Pr > Nd > Er for *A. syrica* roots and Nd > Er > Pr or Nd > Pr > Er for *R. sativus* roots. The mutual relationship between REEs, as well as the influence of other elements present in soil, may be a factor that modulates the higher or lower phytoextraction of particular REEs, which could be another aspect influencing the accumulation of REEs as described, e.g., by Olivares et al. (2014).

When comparing the concentration of particular REEs in soil and in the earth’s crust (EPA 2012), a significantly higher concentration of Tm and Y was observed in the studied soil near the road. In the case of the rest of the REEs, their concentration was many times lower than in the earth’s crust (Wedepohl 1995). The use of selected REEs such as Ce, La, or Nd in automobile converter catalysts used to enhance pollutant oxidation, explains the high concentration, particularly of La and Ce in road dust (Djingova et al. 2003) but also in soil near frequented roads (Figueiredo et al. 2009; Mikołajczak et al. 2017). Figueiredo et al. 2009 studied soils in 14 public parks of São Paulo and found the following concentration of elements: Ce > La > Nd > Yb = Sm > Tb = Lu = Eu, while in our studied soil the concentration was as follows: Ce > Nd > La > Yb > Tb =Lu = Eu > Sm. The differences in the concentration of particular REEs may be related not with density of traffic but the natural geological composition of the soils. The concentration of REEs in soil was different with respect to the distance from the road. This suggests the important role of traffic in soil contamination. In spite of the fact that some authors have found no concise correlation between REE concentrations in soil and plants (Tyler 2004; Wiche and Heilmeier, 2016), we have stated such a relationship. It is likely that the differences between our results and those of the above mentioned authors were due to the amount of bioavailable forms of REEs and also the pH and Eh of soils which significantly influenced REE phytoextraction (Cao et al. 2000). In many cases, other authors have found the same tendency in trace element deposition (Kafoor and Kasra 2014), where the concentration of bioavailable elements for plants was lower the further away from the road (Çolak et al. 2016). Xinde et al. (2000) pointed out the necessity for chemical fractionation and multiple regression analysis to estimate the bioavailability of REEs and to indicate differences in the bioavailability of particular species of REEs of individual elements. In our paper, such an analysis was not done; therefore, a crucial role is played by pH or redox potential (Cao et al. 2001) of soil related with mobility (or not) and electric conductivity, being an indicator of the presence of additional stress for plant (salinity). The pH of the soil analyzed in our experiment was 6.00–6.05 with visible uptake of REEs confirmed by BCF > 1. Thomas et al. (2014) found clear differences in the phytoextraction of selected REEs (Ce, La, Y) by native Canadian plant species and commonly used crop species in terms of different pH values (4.08 and 6.74). A higher pH value was related to a generally lower phytoextraction of REEs, which suggests that the studied herbaceous plant species could be able to uptake these elements more effectively in the case of more acidic soils. It is worth underlining that a small change in the pH of soil may be related with significant differences in REE phytoextraction, as described by Wiche et al. (2016). The authors have shown that phytoextraction of La and Nd was significantly higher in herbs than in grasses and REEs are more effectively accumulated in slightly acidic than slightly alkaline soils. On the other hand, the results of the study by Khan et al. (2017), which analyzed plants of the *Cyperaceae*, *Gleicheniaceae*, and *Melastomataceae* families and their potential for phytoextraction of REEs, indicated that this process is also EC dependent. Moreover, lower pH and EC values were related to higher concentrations of, e.g., Ce and Y, while the concentration of, e.g., La and Sc was lower. This observation is similar to the relationships described by Thomas et al. (2014), and it can explain the relationships observed in our studies. It is worth underlining that the higher salinity (higher EC values) in our studies compared to those in the paper of Khan et al. (2017) pointed to the high potential of the studied herbaceous plant species uptake of REEs from soils near roads.

We have shown that concentration of REEs (LREEs and HREEs) in soils decreased with distance from the road, and the same tendency was observed for the content of REEs in total plant biomass. Interestingly, no such correlation was observed for certain LREEs and the majority of HREEs. Wiche et al. (2017) have also found differences in the concentration of both groups of elements in soil and, similarly to our results, the efficiency of the phytoextraction of LREEs was higher than that of HREEs. Very similar observations for many other elements (especially As, Cu, Pb, and Zn) were described by Pivić et al. (2013), Modlingerová et al. (2012), or Çelenk and Kiziloğlu (2015). The diverse efficiency of phytoextraction of REEs could also be an effect of the changes in the physiology of these plants, such as the biosynthesis of selected low molecular weight organic acids (LMWOAs), especially oxalic, acetic, and citric acids, excluded from the rhizosphere or the creation of phenolic acids (salicylic acid) as a response to oxidative stress caused by trace element occurrence (unpublished data for presented plant species). Wiche et al. (2017) underlined recently the significant role of these acids, especially citric acid, as a factor increasing the mobility of REEs in soil and finally increasing that of the phytoextraction of this group of elements. *A. vulgaris* L. was a species characterized by higher biomass of its root system able to effectively phytoextract trace elements (also REEs), while the ability of *T. officinale* was lower and *T. repens* L. the lowest among the studied plant species. The root systems of all the plant species were found at 0–15 cm depth, where characteristics of soils for plants growing at the same distance from the road were the same. For this reason, differences in the creation of selected LMWOAs by particular herbaceous plant species are responsible for higher or lower phytoextraction of REEs, as previously described by Shan et al. (2003). Another important fact relating to the diverse distribution of REEs in soils and phytoextraction (Fig. 2) is that traffic is likely to be an important source of REEs with limitation of their delivery to further distances from the road (Li et al. 2010).

We know that the plants studied in this paper were able to phytoextract REEs but how efficient was this process in relation to other plants? The described efficiency of REE phytoextraction is significantly lower than that for hyperaccumulators such as *Dicropteris dichotoma* (Shan et al. 2003), where concentration of La, Ce, Nd, and Pr was up to 0.7% of its dry leaf biomass. Also Khan et al. (2017) have described potential of plant species belonging to the *Cyperaceae, Gleicheniaceae*, and *Melastomataceae* families to phytoextract REEs with very high values of BCF (12.4–151.7). The response of the selected plant species may be also be different as regards their specific environmental requirements, such as the amount of other trace elements important for their growth, which in the case of REEs is especially important. Wang et al. (2008) studies on horseradish have shown that there is a relationship between REEs and the concentration of other trace elements. The growth of the studied herbaceous plant species near the frequented road with many other plant species had to be related to mutual interactions that modified the efficiency of REE uptake. The importance of the influence of such relationships (plant growth stimulation or inhibition, synergism or antagonism between elements) both for REEs and many other elements and plants was shown by Wiche et al. (2016), Liu et al. (2017), or Drzewiecka et al. (2017). Moreover, this relation can influence the growth conditions. According to Chen et al. (2016), *Dicranopteris dichotoma* is a plant recommended for controlling REE migration which can be related with intensified phytoextraction of these elements just by this plant species. It is known that a low concentration of REEs in soil is usually related to plant growth stimulation, while high concentrations have a negative influence for both plant growth and phytoextraction of trace elements (Zhang et al. 2013), but there are limited data about the influence of all 17 REEs to plant response (de Oliveira et al. 2015), which makes a clear comparison of the REEs phytoextraction potential of different plant species impossible. Even the comparison of BCF values is not enough as regards the high amount of environmental factors that influence REEs phytoextraction in plants, therefore promising plants for phytoremediation (e.g., *Helianthus annuus*) can be characterized by BCF < 1 (Kötschau et al. 2014).

The plants studied in this paper are commonly found in the vicinity of roads. The biomass of these plants is many times higher than many other plant species growing individually near roads. For this reason, phytoextraction of numerous elements (including REEs) can be highly effective. Although there is clear evidence of the harmful influence of REEs on human health (Pagano et al. 2015a) and plant development (Zhang et al. 2013), the authors have no access to literature where similar studies can be found. On the other hand, there are some data that describe or compare the herbaceous plant species tested in our study. Malinowska et al. (2015) revealed that *Taraxacum* spec. was more effective in Cu and Zn phytoextraction than *Achillea millefolium* L., *Rumex acetosa* L., or *Vicia cracca* L. independently of the distance from the road. Diatta et al. (2003), who analyzed *T. officinale*, showed this plant species to be a bioindicator of soil contamination. Both the high efficiency of element phytoextraction and negative changes in leaf anatomy (changes in morphology) were described by Bini et al. (2012), who found that this species is highly effective in the phytoextraction of toxic elements, in both roots and stems (high values of translocation factor). A great affinity for effective phytoextraction of many other elements was also described by Bech et al. (2016), who showed that *T. officinale* can be characterized by diverse translocation of As, Pb, and Zn in its plant organs (roots, stem) depending on the mineral composition of soils. Based on these findings, we can assume that this species would also possess high phytoextraction potential for other elements, such as REEs. This was confirmed in our paper by the higher phytoextraction of REEs observed in this species compared to *A. vulgaris*. Durães et al. (2014) did not find any correlation between the concentration of REEs in the rhizosphere and in plants, which suggests that transport of REEs from the environment to the plant can occur in other ways. For this reason, the relationships described in our paper between phytoextraction of REEs in particular plant organs, generally in accordance with those presented by Durães et al. (2014), are characteristic of the analyzed area. To confirm the same relationships between tested plant species would require further analysis in other ecosystems. Nevertheless, the high efficiency of REE phytoextraction by *T. officinale* F.H. Wigg and *T. repens* L. in particular may be characteristic of the greater ability of these plant species in relation to other elements, as presented, e.g., by Porębska and Ostrowska (1999).

## Conclusion

The development of new technologies that use REEs is the main cause of their transport to the environment. Currently, our knowledge of the distribution of REEs (especially LREEs and HREEs) and their phytoextraction from the soils in the vicinity of roads is very limited. The results of this paper confirm that the concentration of REEs decreases with distance from the road, which was the expected conclusion. On the other hand, differences in REEs phytoextraction among some of the most commonly occurring roadside plants underlined their potential to decontaminate soils. All three herbaceous plant species are small with low biomass but they usually grow in dense groups of specimens per size area. The most effective, *T. repens* L., may be especially useful for phytoextraction purposes but the other two plant species are, in our opinion, promising and would merit further investigation. Additionally, it is worth emphasizing that to determine an ecological problem of the presence of REEs in the environment it is necessary, and sufficient, to conduct an analysis of Ce, Er, and Nd concentration.
